# 82.5 GHz Photonic W-Band IM/DD PS-PAM4 Wireless Transmission over 300 m Based on Balanced and Lightweight DNN Equalizer Cascaded with Clustering Algorithm

**DOI:** 10.3390/s25195986

**Published:** 2025-09-27

**Authors:** Jingtao Ge, Jie Zhang, Sicong Xu, Qihang Wang, Jingwen Lin, Sheng Hu, Xin Lu, Zhihang Ou, Siqi Wang, Tong Wang, Yichen Li, Yuan Ma, Jiali Chen, Tensheng Zhang, Wen Zhou

**Affiliations:** 1 State Key Laboratory of Integrated Chips and Systems, Fudan University, Shanghai 200433, China; 23210720159@m.fudan.edu.cn (J.G.); 23210720305@m.fudan.edu.cn (J.Z.); 22210720076@m.fudan.edu.cn (S.X.); 21210720232@m.fudan.edu.cn (Q.W.); 23210720058@m.fudan.edu.cn (J.L.); 23210720170@m.fudan.edu.cn (S.H.); 23210720218@m.fudan.edu.cn (X.L.); 24210720069@m.fudan.edu.cn (Z.O.); 24210720273@m.fudan.edu.cn (S.W.); 24210720274@m.fudan.edu.cn (T.W.); 23210720197@m.fudan.edu.cn (Y.L.); 22210720195@m.fudan.edu.cn (Y.M.); 23210720141@m.fudan.edu.cn (J.C.); 23210720309@m.fudan.edu.cn (T.Z.); 2 School of Future Information Science and Technology, Fudan University, Shanghai 200433, China

**Keywords:** 6G communication, clustering algorithm, neural networks, nonlinear compensation, SMOTE, terahertz technology

## Abstract

With the rise of 6G, the exponential growth of data traffic, the proliferation of emerging applications, and the ubiquity of smart devices, the demand for spectral resources is unprecedented. Terahertz communication (100 GHz–3 THz) plays a key role in alleviating spectrum scarcity through ultra-broadband transmission. In this study, terahertz optical carrier-based systems are employed, where fiber-optic components are used to generate the optical signals, and the signal is transmitted via direct detection in the receiver side, without relying on fiber-optic transmission. In these systems, deep learning-based equalization effectively compensates for nonlinear distortions, while probability shaping (PS) enhances system capacity under modulation constraints. However, the probability distribution of signals processed by PS varies with amplitude, making it challenging to extract useful information from the minority class, which in turn limits the effectiveness of nonlinear equalization. Furthermore, in IM-DD systems, optical multipath interference (MPI) noise introduces signal-dependent amplitude jitter after direct detection, degrading system performance. To address these challenges, we propose a lightweight neural network equalizer assisted by the Synthetic Minority Oversampling Technique (SMOTE) and a clustering method. Applying SMOTE prior to the equalizer mitigates training difficulties arising from class imbalance, while the low-complexity clustering algorithm after the equalizer identifies edge jitter levels for decision-making. This joint approach compensates for both nonlinear distortion and jitter-related decision errors. Based on this algorithm, we conducted a 3.75 Gbaud W-band PAM4 wireless transmission experiment over 300 m at Fudan University’s Handan campus, achieving a bit error rate of 1.32 × 10^−3^, which corresponds to a 70.7% improvement over conventional schemes. Compared to traditional equalizers, the proposed new equalizer reduces algorithm complexity by 70.6% and training sequence length by 33%, while achieving the same performance. These advantages highlight its significant potential for future optical carrier-based wireless communication systems.

## 1. Introduction

As the era of 6G approaches, the global demand for high-speed, low-latency, and large-capacity wireless communications continues to grow [1,2,3,4,5,6,7,8]. The terahertz (THz) frequency band (0.1–3 THz), with its ultra-wide bandwidth, excellent directivity, and short wavelength, is widely regarded as a core enabling technology for 6G communications. In particular, within large-scale, multi-band wireless transmission systems, terahertz communications can meet the demands for massive data transfer and low latency, making it an essential component of next-generation mobile communication systems. However, despite its theoretically ultra-high transmission capacity, terahertz communications still face numerous challenges in practical applications, especially performance bottlenecks arising from signal nonlinearities and low signal-to-noise ratios.

Generally, there are two primary methods for generating terahertz waves: electrical and optical methods. However, when using electrical methods to generate terahertz signals, the transmission speed is limited by the bandwidth constraints of electrical equipment. In contrast, optical methods for generating broadband terahertz radio signals have been recognized as a key solution for mobile communications. The advantages of optical methods include broader bandwidth, higher modulation efficiency, and lower harmonic interference, making them well-suited for high-speed terahertz communication. We will incorporate this explanation into the manuscript to better clarify the relationship between terahertz radio transmission and the Radio over Fiber approach.

Specifically, the common nonlinear effects in terahertz communication systems primarily originate from high optical power in optical fibers, optoelectronic devices such as photodiodes, high-power amplifiers (HPAs) in wireless channels, and nonlinear impairments in downconverters [9,10,11,12,13,14]. These nonlinearities significantly degrade signal quality and transmission efficiency, while signal attenuation and noise interference become particularly severe in long-distance, high-speed wireless transmissions. Although conventional digital signal processing (DSP) techniques can effectively mitigate issues such as dispersion and frequency offset, they are limited in addressing the strong nonlinear effects and the low signal-to-noise ratio in terahertz systems. In high-speed, large-capacity terahertz communications, the constraints of conventional DSP algorithms in nonlinear compensation become increasingly evident, thereby hindering the full exploitation of the potential advantages of terahertz signals [15,16,17].

To address these challenges, nonlinear equalization techniques based on machine learning methods such as deep neural networks (DNNs) have emerged as a prominent research focus in the field of terahertz communications in recent years. Architectures such as deep neural networks and convolutional neural networks can be trained on large datasets to automatically learn and extract the nonlinear features of signals, thereby achieving more accurate nonlinear compensation [18,19,20]. To further enhance the capacity of modulation-constrained channels, probabilistic shaping techniques have been introduced into optical fiber communication systems [21,22,23]. However, the introduction of PS may lead to an imbalanced data distribution, thereby reducing the generalization capability of the model. An imbalanced training dataset can lead to insufficient learning of minority classes during the machine learning process, thereby degrading the overall system performance. Previous studies have shown that this issue can be mitigated by performing random resampling of the training dataset, including both undersampling and oversampling. Undersampling reduces the number of majority class samples, potentially causing the loss of useful information and reducing system capacity, whereas oversampling increases the number of minority class samples and can effectively address the data imbalance problem. As an oversampling method, the Synthetic Minority Oversampling Technique (SMOTE) generates new minority-class samples in the feature space, thereby balancing the dataset structure and alleviating the training difficulties caused by data imbalance [24,25,26,27,28,29,30,31,32]. This approach produces representative minority-class samples, thereby avoiding performance degradation due to insufficient minority-class data.

Furthermore, direct detection systems are highly susceptible to optical multipath interference (MPI) noise [33,34]. MPI noise is one of the primary performance impairments in direct detection links, originating from multiple reflections at contaminated fiber connectors. The reflected signals exhibit random optical delays, which, after direct detection, convert laser phase noise into signal-dependent intensity noise through interference, thereby increasing the bit error rate (BER) of the system. At the receiver, MPI impairment often manifests as step-like amplitude variations or jitter in the signal, which is particularly pronounced in multi-level modulation formats such as PAM4. Such jitter can render fixed decision thresholds ineffective, thereby limiting the performance of conventional deep neural network equalizers, with constellation diagrams still exhibiting significant amplitude spread even after equalization. To address this issue, we incorporate a clustering algorithm into the DNN equalizer to assist the decision-making process. In pilotless terahertz radio-over-fiber (RoF) systems, receiver-side equalization constitutes a typical blind equalization problem. For PAM4 signals exhibiting four distinct amplitude clusters, the clustering algorithm can automatically identify the cluster structure and perform self-supervised adaptive threshold updates. This method maintains decision stability under signal level jitter while offering low complexity, reducing the DNN’s dependence on complex network architectures. As a result, it enables a lightweight design, enhancing feasibility and efficiency in practical deployments.

In this paper, we propose a lightweight neural network equalizer for terahertz radio-over-fiber systems, integrating the Synthetic Minority Oversampling Technique and a clustering algorithm to address training data imbalance and signal amplitude jitter, respectively. SMOTE significantly enhances the training performance of the equalizer by balancing the data distribution, while the clustering algorithm adaptively optimizes decision thresholds based on the amplitude distribution of the received signals after equalization, thereby improving decision accuracy and system robustness. In a 3.75 Gbaud W-band PAM4 wireless transmission experiment, the proposed method achieved a bit error rate of 1.32 × 10^−3^ over a transmission distance of 300 m, corresponding to approximately a 70.7% reduction in BER compared with conventional DNN equalizers. Furthermore, the proposed scheme outperforms conventional equalizers in terms of training accuracy, training data requirements, and computational complexity, thereby demonstrating superior engineering feasibility and computational efficiency. Experimental results indicate that the proposed scheme holds great potential for future optical carrier-based 6G wireless communication systems.

## 2. Principles

### 2.1. SMOTE (Synthetic Minority Oversampling Technique)

**Figure 1:**
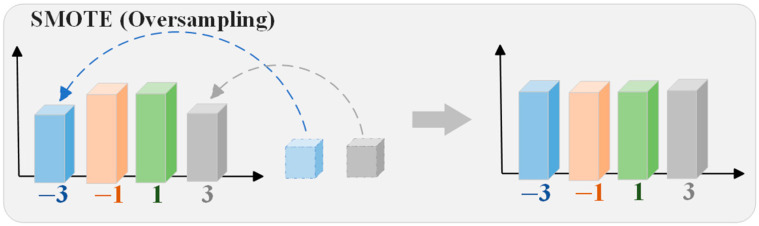
The principle of SMOTE.

In neural network (NN) classification algorithms, a class imbalance issue arises when the sample size of certain classes is significantly larger than that of others. We define the class with a larger sample size as the majority class, while the class with fewer samples is defined as the minority class. For long-range, high-speed wireless channels affected by nonlinearity, phase noise, and high loss, learning from minority class samples becomes extremely challenging in the presence of significant class overlap. The main function of the SMOTE algorithm is to overcome the class imbalance issue by balancing the training dataset. In this paper, we applied the SMOTE at the receiver end (Rx) of the W-band PS-PAM4 wireless optical fiber transmission [35]. The basic principle of SMOTE is illustrated in Figure 1.

The pseudocode of the SMOTE algorithm is shown in Algorithm A1, provided in Appendix A.

The specific steps of the SMOTE algorithm are illustrated in the diagram, and the process is as follows:

Step 1: Input parameters, such as $X_{\mathrm{minority}}$: the minority class sample set, *N*: the number of synthetic samples to generate (which can be a ratio or total number), *k*: the number of nearest neighbors to search for each sample.

Step 2: Create an empty $X_{synthetic}$ set to store the generated synthetic samples.

Step 3: Traverse the minority class sample set $X_{\mathrm{minority}}$, processing each sample $x_{i}$ one by one.

Step 4: Use methods such as Euclidean distance to find the *k*-nearest neighbors of each sample $x_{i}$.

Step 5: Based on the target number of generated samples *N*, perform the corresponding number of operations for each minority class sample $x_{i}$.

Step 6: Generate synthetic samples: Randomly select a sample $x_{neigh}$ from the *k*-nearest neighbors of the current sample $x_{i}$, and calculate the difference vector *d*:

(1) $$d=x_{neigh}-x_{i}$$

Generate a random number $\lambda \in \left[0,1\right]$, and generate new synthetic samples through interpolation.

Step 7: Repeat Steps 3 to 6 until all samples are processed. SMOTE increases the number of minority-class samples, thereby making the class distribution in the dataset more balanced.

**Figure 2:**
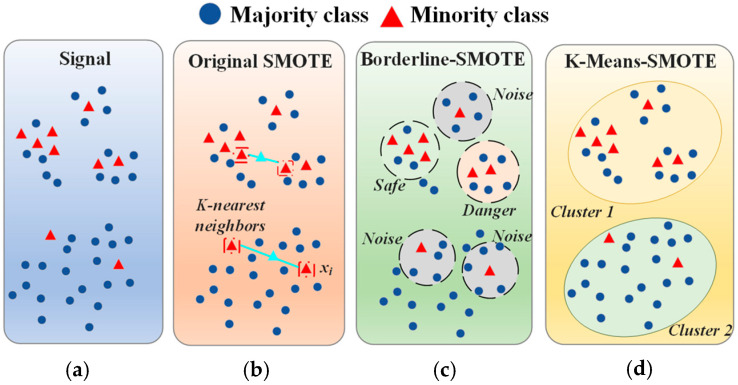
The principle of different types of SMOTE algorithms (**a**) Original signal, (**b**) Original SMOTE algorithm principle, (**c**) Borderline-SMOTE algorithm principle, (**d**) K-Means SMOTE algorithm principle.

SMOTE, Borderline-SMOTE, and K-Means SMOTE are three common oversampling techniques used to address class imbalance by generating synthetic minority-class samples [36,37,38]. While they share a similar goal, each method works differently and focuses on different aspects, as illustrated by their underlying principles in Figure 2.

SMOTE is a basic oversampling method that generates new synthetic samples by performing linear interpolation between minority class samples. For each minority class sample, SMOTE randomly selects a neighbor from its *k*-nearest neighbors and generates a new sample based on the distance between the two samples. This method is simple and intuitive, but may result in unreasonable synthetic samples, especially when the minority class sample distribution is sparse. The generated synthetic samples may not align with the original data, which can affect the accuracy of the model.

Borderline-SMOTE is an improvement to SMOTE, focusing on the part of the minority class samples near the decision boundary. It divides the minority class samples into two categories: safe and dangerous points. Safe points are located far from the decision boundary and generally do not require oversampling, while dangerous points are near the decision boundary and are crucial to the classifier’s decision. Borderline-SMOTE oversamples only the dangerous points, rather than oversampling all minority class samples. In this way, it more effectively enhances the model’s understanding of the decision boundary, improving classification performance. However, this method may increase the complexity of the decision boundary, especially when there are many noisy points, potentially causing the model to learn the boundary incorrectly.

K-Means SMOTE combines the K-Means clustering algorithm with SMOTE. It first clusters the minority class samples into multiple clusters using K-Means and then generates synthetic samples within each cluster. This way, K-Means SMOTE ensures that the newly generated synthetic samples lie within the dense areas of the clusters, rather than being randomly distributed. This ensures that the generated synthetic samples better match the distribution of the original data, preserving the local structure of the data. While this method can improve the quality of synthetic samples, it requires clustering first, which increases computational complexity, and the clustering results may be affected by the number of clusters and the choice of initial centroids.

In summary, while the Synthetic Minority Oversampling Technique (SMOTE), Borderline-SMOTE, and K-Means SMOTE share the objective of mitigating class imbalance through synthetic sample generation, they differ markedly in their strategies and applicability. SMOTE offers a basic yet potentially coarse-grained approach; Borderline-SMOTE enhances the sensitivity to the decision boundary but may be prone to noise amplification; and K-Means SMOTE enhances fidelity to the original data distribution at the expense of increased computational complexity. The selection of an appropriate method should be guided by the specific characteristics of the dataset and the trade-offs among model robustness, boundary precision, and algorithmic efficiency.

### 2.2. DBSCAN (Density-Based Spatial Clustering of Applications with Noise)

DBSCAN (Density-Based Spatial Clustering of Applications with Noise) is a density-based clustering algorithm. It identifies cluster structures based on the density of data points, making it particularly suitable for handling data with noise and irregularly shaped clusters. Unlike many other clustering algorithms, DBSCAN does not require the prior specification of the number of clusters. Instead, it automatically identifies clusters in the data based on density and effectively distinguishes noise points [39,40,41,42,43,44,45].

The basic concept of DBSCAN is to define clusters using the following three types of data points:

Core point: If a data point’s neighborhood (within a specified radius ε) contains at least MinPts points, the point is considered a core point. A core point represents the center of a cluster and can assign surrounding sufficiently dense points to the same cluster.

Border point: If a point’s neighborhood contains fewer than MinPts data points, but the point lies within the neighborhood of a core point, it is considered a border point. Border points do not form new clusters but belong to a cluster defined by a core point.

**Figure 3:**
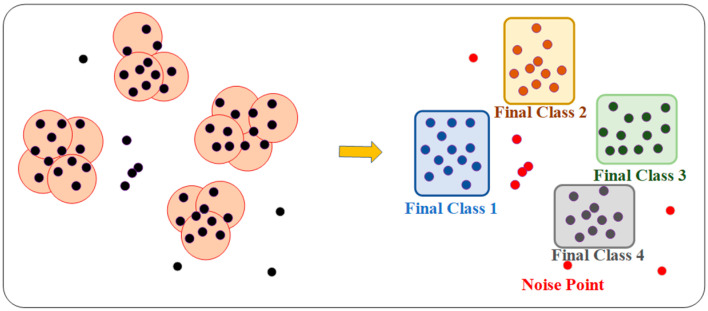
The principle of DBSCAN.

Noise point: If a point is neither a core point nor located within the neighborhood of any core point, it is considered a noise point and typically not assigned to any cluster. The basic principle of DBSCAN is depicted in Figure 3.

The pseudocode of the DBSCAN algorithm is shown in Algorithm A2, provided in Appendix B.

The specific steps of the DBSCAN algorithm are depicted in the diagram below, and the process is as follows:

Step 1: Input parameters, including the set of all data points *D*, ε (Epsilon), and MinPts. ε represents the radius of the neighborhood, which defines the range of a data point’s neighborhood. MinPts is the minimum number of points required to form a core point, meaning that a point must have at least MinPts points within its neighborhood to be considered a core point.

Step 2: Initialization: Set ClusterId to 0 to track the current cluster number. Mark all points as unvisited and initialize each point’s cluster label, ClusterLabels[i], as None.

Step 3: Traverse each unvisited data point *P*, mark *P* as visited, and find all points within *P*’s ε-neighborhood, forming the set NeighborPts, i.e., all points within distance ε of *P*. Then, determine if *P* is a core point. If the number of points in NeighborPts is less than MinPts, mark *P* as a noise point; otherwise, create a new cluster (ClusterId = ClusterId + 1) and mark *P* as a member of the current cluster.

Step 4: Expand the cluster. For each neighbor point *Q* of *P*, if *Q* is unvisited, mark *Q* as visited and find the points in *Q*’s ε-neighborhood, forming the set NeighborPts’. If the number of points in NeighborPts’ is greater than or equal to MinPts, *Q* is also a core point, and the points in NeighborPts’ are added to the current cluster’s expansion queue, NeighborPts; if *Q* has not been assigned to any cluster (ClusterLabels[*Q*] is None), assign *Q* to the current cluster: ClusterLabels[*Q*] ← ClusterId.

Step 5: Repeat the above operations until all points are visited.

### 2.3. DNN Equalizer

**Figure 4:**
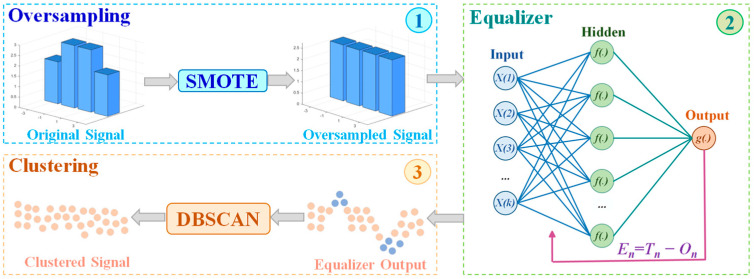
Principle Diagram of DNN Equalizer Combining SMOTE and DBSCAN. (1) Oversampling part, (2) Equalizer part, (3) Clustering part.

In this paper, we employ a DNN equalizer to handle nonlinear distortions, with the modulated signal being a PAM signal. During training, we fed a portion of the original dataset as training data into the network. Finally, the bit error rate decision was made based on the equalized test signal. It is worth noting that the SMOTE algorithm, as a data preprocessing method, is applied before the DNN equalizer, while the DBSCAN clustering algorithm, as a data postprocessing method, is applied after the DNN equalizer. With the help of both, the DNN can achieve better bit error performance with reduced complexity, and the overall workflow alongside the principles of SMOTE and DBSCAN are depicted in Figure 4.

The real-valued DNN equalizer is a signal processing technique based on deep neural networks, primarily used to overcome signal distortion caused by factors such as nonlinearity and noise in communication systems. The core idea of this equalizer is to train a deep neural network to learn the complex features of the signal, thereby recovering the distorted signal. The DNN equalizer proposed in this paper consists of an input layer, hidden layers, and an output layer. The real-valued DNN equalizer is a network composed of multiple layers of neurons. Each layer processes the input signal through an activation function and passes the output to the next layer. The number of layers and the number of neurons in each layer determine the network’s capacity and complexity. For the real-valued DNN equalizer, both the input and output data are real numbers, and all neurons, weights, and activation functions are also based on real numbers. The input vector is represented as $X\left(n\right)={\left[x\left(n\right),x\left(n-1\right)\ldots ,x\left(n-N_{0}+1\right)\right]}^{T}$, This represents a memory window of length $N_{0}$, where $X_{0}\left(n\right)$ represents the current value of the input signal, while $X_{0}\left(n-1\right)$ to $X_{0}\left(n-N+1\right)$ represent the previous signal values. This is performed to ensure that the network can handle temporal changes in the signal and take into account historical information to help recover the signal affected by nonlinearity.

The input vector $X\left(n\right)$ is passed to the first layer of the neural network and processed through the weighted connections in the hidden layers. At each hidden layer, the input signal is multiplied by the weights $w_{i_{l-1}j_{l}}^{l}$ of that layer and processed through a nonlinear activation function. *i* represents the *i*-th neuron in the previous layer (Layer *L* − 1), and *j* represents the *j*-th neuron in the current layer (Layer *L*). Thus, each part of the input signal (such as the real and imaginary parts) undergoes a weighted sum and nonlinear transformation at each neuron in the neural network. 

After passing through each hidden layer, the output of the neurons is passed on to the next layer. To introduce more complex feature representations, the network structure uses multiple hidden layers. These hidden layers extract different levels of signal features by applying nonlinear transformations (such as ReLU or Sigmoid) to the input signal. Considering the issues of gradient explosion and gradient vanishing, we chose “ReLU” as the activation function, which can be described as

(2) f(x)=relu(x)=max(0,x)

After l hidden layers, the sum of the outputs from different nonlinear neurons is

(3) $$O_{n}=h_{j_{L}}^{L}=f\left(\sum_{i_{L-1}}^{N_{L-1}}w_{i_{L-1}j_{L}}^{L}\cdot f\left(\sum_{i_{L-2}}^{N_{L-2}}w_{i_{L-2}j_{L}-1}^{L-1}\ldots f\left(\sum_{i_{1}=1}^{N_{1}}w_{i_{1}j_{2}}^{2}\cdot f\left(\sum_{i_{0}=1}^{N_{0}}w_{i_{0}j_{1}}^{1}x_{i}\right)\right)\right.\right.$$

Here, *L* represents the current layer number, $h_{j_{L}}^{L}$ represents the output of the *j*-th neuron in layer *L*, f represents the nonlinear activation function, $w_{i_{L-1}j_{L}}^{L}$ represents the weights of layer *L*: the weights from the $i_{L-1}$ neuron in the previous layer to the $i_{L}$ neuron in the current layer, and $N_{L}$ represents the number of neurons in layer *L*. The weights are adaptively updated based on the mean squared error (MSE) loss function, with the update process shown as follows:

(4) $$E_{n}=T_{n}-O_{n}$$

(5) $$J_{n}=\frac{1}{2}\sum_{n}^{N}{\left(E_{n}\right)}^{2}$$

(6) $$w_{ij}^{k+}=w_{ij}^{k}-\alpha \frac{\partial J_{n}}{\partial w_{ij}^{k}}$$

Here, $T_{n}$ represents the transmitted signal, $O_{n}$ represents the output signal of the network, $E_{n}$ represents the error signal, and $J_{n}$ represents the cost function. The output result $O_{n}$ is subtracted from the expected output value $T_{n}$ to obtain the error value $E_{n}$, which is then fed back to the training equalizer for further calculation. Using the algorithm, the weights $w_{i_{j}}^{l}$ are updated continuously until the preset epoch or error value is reached.

For the training configuration, we use the mean squared error (MSE) loss function to measure the deviation between the predicted output and the true values. The Adam optimizer is employed to adaptively adjust the learning rate to enhance convergence efficiency, with an L2 regularization term introduced to suppress overfitting. The weight decay factor is set to 1 × 10^−8^. The initial learning rate is set to 0.001, and a StepLR learning rate scheduler is used, which reduces the learning rate by half every 10 epochs to enhance model stability and generalization capability.

## 3. Experimental Setup and Link Budget

### 3.1. Experimental Setup

**Figure 5:**
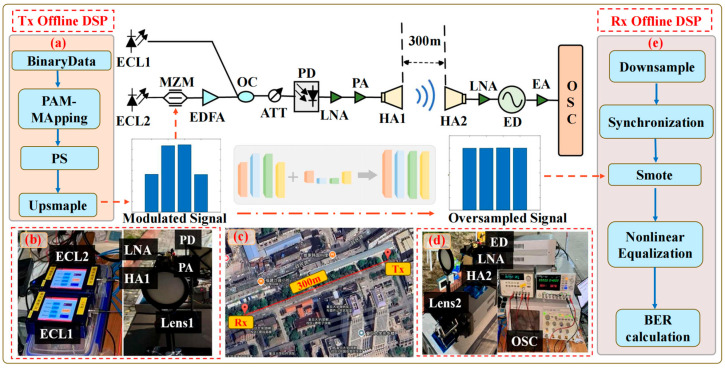
Experimental setup for W-band PAM transmission in a 300 m free-space wireless transmission system and photos of (**a**) the block diagram of Tx DSP; (**b**) the transmitter side; (**c**) 300 m wireless link; (**d**) the receiver side; (**e**) the block diagram of Rx DSP.

Figure 5 shows the experimental setup for W-band PAM signal transmission over a 300 m free-space wireless link. Figure 5a presents the block diagram of the transmitter-side digital signal processing (Tx DSP). In this module, the baseband PS-PAM4 signal is generated offline using MATLAB R2024b software and prepared for optical modulation.

Figure 5b shows the transmitter-side hardware. The generated baseband signal is modulated using a Mach–Zehnder modulator (MZM) with a 20 GHz optical bandwidth and a 6 dB insertion loss. The coupled optical beam is adjusted by an attenuator (ATT) to achieve the optimal input power for the photodetector (PD). The PD converts the optical signal into a W-band electrical signal, which is then amplified by a power amplifier (PA) for transmission. External cavity laser 1 (1550.68 nm, 100 kHz linewidth, average power 9.5 dBm) and external cavity laser 2 (1550 nm, 100 kHz linewidth, average power 13.5 dBm) are employed as optical sources. They are combined through a polarization-maintaining optical coupler with a frequency separation of 85 GHz to enable photon-heterodyne generation of the terahertz signal. In this experiment, the PD operates at a −2 V DC bias with a frequency range of 10–170 GHz, and its input optical power is maintained between −0.5 dBm and 2.5 dBm via the ATT. The PA has a gain of 30 dB; however, due to preamplification by a 30 dB low-noise amplifier (LNA, NF = 4 dB), it occasionally operates in saturation, thereby introducing nonlinear distortion.

Figure 5c depicts the 300 m free-space wireless link. The generated 82.5 GHz signal is radiated through a transmitting W-band horn antenna (HA, gain 25 dB) precisely positioned at the focal point of lens 1 (diameter 10 cm, focal length 20 cm). A second identical lens focuses the beam onto the receiving HA. The lens pair provides an additional gain of 20 dB. Without this optical focusing, a simple horn-to-horn link would not function properly due to low SNR in the absence of sufficient W-band amplification.

Figure 5d shows the receiver-side hardware. The received 82.5 GHz signal is first amplified by an LNA with 30 dB gain, then demodulated by an envelope detector to obtain the baseband signal. The baseband output is further amplified by an electrical amplifier (EA) with a gain of 14.4 dB (NF = 5.4 dB) before being captured by a real-time oscilloscope with a 50 GHz sampling rate.

Figure 5e illustrates the receiver-side DSP chain (Rx DSP). The captured waveform undergoes offline processing, including downsampling, synchronization, and nonlinear equalization, to recover the transmitted data and evaluate system performance. The key parameters of the main experimental components are summarized in Table 1.

In the photon-assisted millimeter-wave system, several key devices at the transmitter—namely the driver amplifier (EA), Mach–Zehnder modulator (MZM), and photodetector (PD)—introduce nonlinear distortions due to their physical nature, which severely limits system performance, especially in high-order modulation formats [46,47].

The nonlinearities of the driver amplifier (EA) primarily manifest as gain compression effects. When the amplifier operates in the linear region, its gain remains constant; however, once the input power exceeds a certain threshold, the amplifier enters saturation, causing a decrease in gain and resulting in nonlinear distortion in the output signal. This effect is particularly significant for amplitude-modulated signals, as it non-uniformly compresses the spacing between different amplitude levels in the constellation diagram. Typically, higher amplitude signals experience more compression. High-order modulation formats, with their denser constellation structures, are more sensitive to nonlinear distortions, often leading to performance degradation even at lower input power levels, with the optimal operating point shifting toward lower input powers.

The nonlinearity of the Mach–Zehnder modulator (MZM) arises from its inherent cosine transfer characteristic, where the output optical field has a nonlinear relationship with the driving voltage:

(7) $$E_{out}\left(t\right)=E_{in}\left(t\right)\cdot \cos\left(\frac{V_{driver}+V_{bias}}{2V_{\pi }}\right)$$

Although MZM generally operates in the region of its transfer function that approximates linearity, the nonlinear distortion is not the primary issue in practical systems due to the limitations of the driver amplifier output range. However, drift in the bias point can introduce significant signal degradation. Theoretically, MZM should be biased at the quadrature point to achieve optimal linearity. However, factors such as temperature drift, aging, and control errors lead to deviations from the theoretical bias voltage, which introduces a DC component, causing overall shift and distortion in the constellation diagram and reducing the system’s signal-to-noise ratio (SNR).

The nonlinearity of the photodetector (PD) is primarily caused by the space-charge effect. When the incident optical power is too high, the density of photo-generated carriers becomes large enough to alter the internal electric field of the device, which reduces the carrier mobility and decreases the responsivity, leading to saturation. In the saturated state, the output current of the detector exhibits a nonlinear relationship with the input optical power, causing high-amplitude points in the constellation diagram to compress inward and resulting in waveform distortion. Similarly to the driver amplifier, high-order modulation formats are more sensitive to the saturation effects of the photodetector.

In summary, the nonlinear mechanisms of the driver amplifier, modulator, and photodetector vary, but the signal constellation distortion and bit error rate degradation they cause are key factors that limit system performance. In practical system design, in addition to optimizing the operating points of these devices, precisely controlling the modulator’s bias voltage, and properly planning the optical power budget to mitigate nonlinear distortions, advanced nonlinear digital signal processing (DSP) techniques at the receiver end are essential.

Although carefully setting system operating conditions can effectively mitigate nonlinear effects, it is still difficult to completely avoid distortion caused by bias drift, device saturation, and other factors in real-world systems. In such cases, nonlinear equalization algorithms (such as Volterra series-based equalizers or neural network-assisted nonlinear compensation) can mathematically model and reverse compensate for the distortions introduced by nonlinear devices. Therefore, even when the system setup is not in its optimal state, powerful receiver-side DSP can significantly restore signal quality and ensure the reliability of the communication link.

Specifically, the signal baud rate of our system is 3.75 Gbaud, and the modulation format is PAM4, corresponding to a link bit rate of 7.5 Gbps. At the receiver end, to ensure strong error tolerance capability, we have introduced a bit error rate decision threshold mechanism based on FEC (Forward Error Correction), with a 7% error correction overhead. Under this overhead condition, the net bit rate of the system is calculated as

(8) $$Net\;Bit\;Rate=\frac{7.5\mathrm{Gbps}}{1+7\%}\approx 7.01\mathrm{Gbps}$$

The W-band 300 m wireless transmission system is deployed along a link between the Benbei Expressway and the north side of the Danyuan Canteen on the Handan campus of Fudan University. The receiver is located on the north side of Yuan Chengying Building on the Handan campus. According to satellite maps, the precise distance between them is 307 m.

### 3.2. Link Budget

In order to effectively increase the wireless transmission distance while maintaining a high data rate, commercial amplifiers that can be used in high-frequency bands can be used to provide large gain and increase output power [48]. This section performs the power budget of the system based on the Friis formula The received power PR can be expressed as

(9) $$P_{R}=P_{T}+G_{T}+G_{R}-FSPL-L_{\mathrm{am}}$$

where $P_{T}$ is the transmission power, $G_{T}$ is the transmitting antenna gain, $G_{R}$ is the receiving antenna gain, *FSPL* is the free-space loss, $L_{\mathrm{am}}$ is the atmospheric absorption, and

(10) $$FSPL=20\log\frac{4\pi df}{c}$$

where *d* is the wireless transmission distance, *f* is the signal frequency, and *c* is the speed of light.

Table 2 is a summary of the power budget. The transmission power is 13 dBm, the transmitting antenna gain is 37 dBi, and the receiving antenna gain is 55 dBi. According to the table data and substituted into the formula, the FSPL is 116.8 dB, and the atmospheric absorption is 0.7 dB/km × 0.2 km = 0.14 dB. Therefore, the final calculated accepted power is −22.44 dBm, which is very close to our experimental result.

## 4. Experimental Results and Discussions

### 4.1. DBSCAN Parameter Analysis and Performance Validation

**Figure 6:**
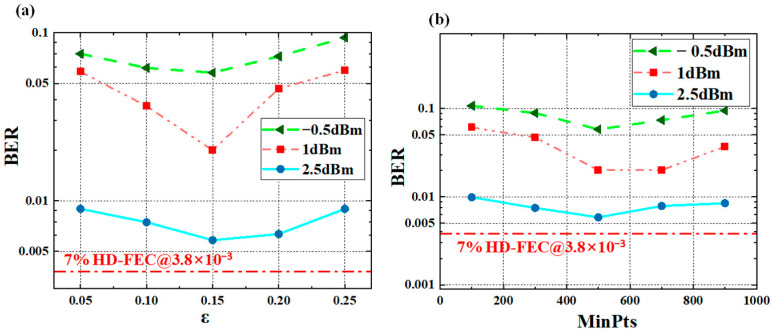
Sensitivity analysis of DBSCAN parameters. (**a**) The impact of ε on BER. (**b**) The impact of MinPts on BER.

Figure 6a shows the relationship between the Bit Error Rate and the neighborhood radius ε at different optical power levels, while Figure 6b illustrates the relationship between BER and MinPts. In this study, we design an adaptive parameter tuning process that integrates the amplitude distribution characteristics of the received signal with clustering visualization effects. First, based on the average amplitude difference between adjacent levels of the PAM4 signal, the search range for ε is fixed within a certain range, while MinPts is set between [100, 900]. A grid search is then performed within this parameter space, and the system’s BER is used to evaluate clustering performance and communication efficiency. The parameter combination that yields the optimal BER and a reasonable clustering structure is selected as the final result. The figures demonstrate that when the optical power is 2.5 dBm, the BER performance is at its best, reaching the lowest value.

The BER curve exhibits a “decreasing, then increasing” trend as the neighborhood radius ε and MinPts increase. This trend, as described, reflects the existence of an optimal ε and MinPts value that minimizes the BER. A small neighborhood radius may result in incomplete clustering, making it difficult to effectively identify signal points, while a large neighborhood radius could lead to excessive merging of clusters, even erroneously including noise points within valid clusters, thus increasing the BER.

**Figure 7:**
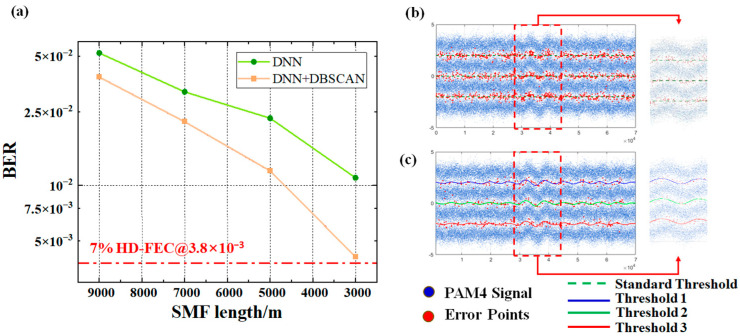
(**a**) BER versus fiber length variation curve; (**b**) Signal after DNN equalization at the receiver; (**c**) Signal after clustering-assisted decision.

An equivalent optical communication system was constructed, and a set of PAM signals was transmitted to simulate the effect of MPI noise on the signal, thereby validating the effectiveness of DBSCAN in mitigating signal jitter. The bit error rate (BER) comparison after processing is shown in Figure 7. The results demonstrate that DBSCAN’s clustering-assisted decision effectively addresses signal jitter, resulting in a notable improvement in BER performance.

### 4.2. Complexity Analysis

**Figure 8:**
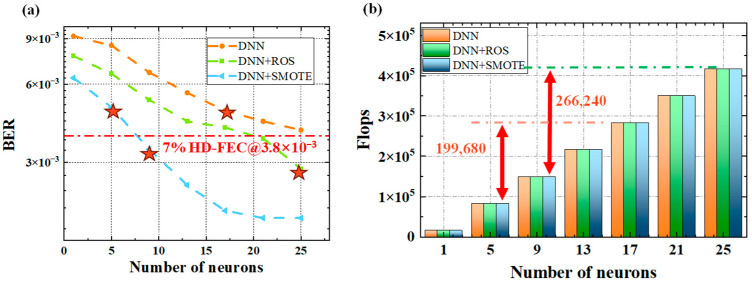
BER and computational complexity for the DNN and the oversampling-enhanced DNN: (**a**) BER versus number of neurons (stars denote points with similar BER); (**b**) FLOPs versus number of neurons.

We investigated the optimal architecture of DNN equalizers for a W-band free-space wireless transmission system over 300 m and conducted a complexity analysis. Each frame contained 32,768 sampled data points, with 10,240 used for training and 22,528 reserved for testing. The input layer of the DNN was configured with 129 neurons. The DNN architecture was fixed to a single hidden layer, and Figure 8a presents the relationship between BER and the number of hidden-layer neurons for the three schemes. As the number of neurons increases, both conventional and enhanced DNN equalizers exhibit decreasing BER, which eventually saturates. Under identical network configurations, the SMOTE-enhanced DNN reduces the average BER by approximately 20.88% compared to the ROS-based DNN (Random Oversampling-based Deep Neural Network) and by 54.75% compared to the standard DNN. Moreover, the SMOTE-enhanced DNN was trained using a stratified K-fold cross-validation protocol, where folds were built before any oversampling to prevent train/test leakage. SMOTE was applied only to the training folds. To validate the physical plausibility of the synthetic samples, we ensured that the generated PAM4 samples preserved amplitude ordering and eye symmetry, avoiding any non-physical distortions. These improvements were further supported by an ablation study comparing the performance of the system with and without SMOTE. The results demonstrate that SMOTE contributes significantly to improving BER performance under the same network conditions.

The computational complexity of the DNN equalizer primarily depends on the depth and width of the network. The number of floating-point operations per second (FLOPs) is directly proportional to the input signal size; here, FLOPs are defined as the computational cost for processing 128 symbols. Overall, the number of FLOPs is linearly proportional to the depth and width of the DNN equalizer. Details of the network complexity are shown in Figure 8b. When the hidden layer contains only one neuron, the single-hidden-layer DNN has a structure of [129-1-1], and the corresponding FLOPs are 16,640. When the hidden layer consists of 17 neurons, the DNN equalizer nearly reaches its optimal performance, with a FLOPs count of 282,880. With 25 hidden neurons, the FLOPs increase to 416,000.

The DNN equalizer augmented with SMOTE enables a reduction in the number of hidden-layer neurons required, thereby markedly decreasing computational complexity. Here, we focus on the test dataset. When the network is constrained to a single hidden layer and operated at comparable complexity, the conventional DNN employs 17 neurons, corresponding to 282,880 FLOPs. In contrast, the SMOTE-assisted DNN equalizer requires only 5 neurons, resulting in 83,200 FLOPs—a reduction of 199,680 FLOPs, equivalent to an approximate 70.6% decrease in computational complexity. Likewise, for DNN equalizers assisted by ROS and SMOTE, the number of hidden-layer neurons is 9 and 25, respectively, with FLOPs decreasing from 416,000 to 149,760, representing a reduction of 266,240 FLOPs and a computational complexity decrease of approximately 63.8%. These results indicate that, for comparable bit error performance, the SMOTE-assisted DNN equalizer offers a more substantial advantage in terms of computational efficiency, particularly in reducing the number of FLOPs. Overall, the findings demonstrate that the integration of SMOTE not only enhances bit error performance but also confers significant benefits in lowering computational complexity, thereby reducing the real-time inference overhead and improving suitability for hardware-constrained implementations.

**Figure 9:**
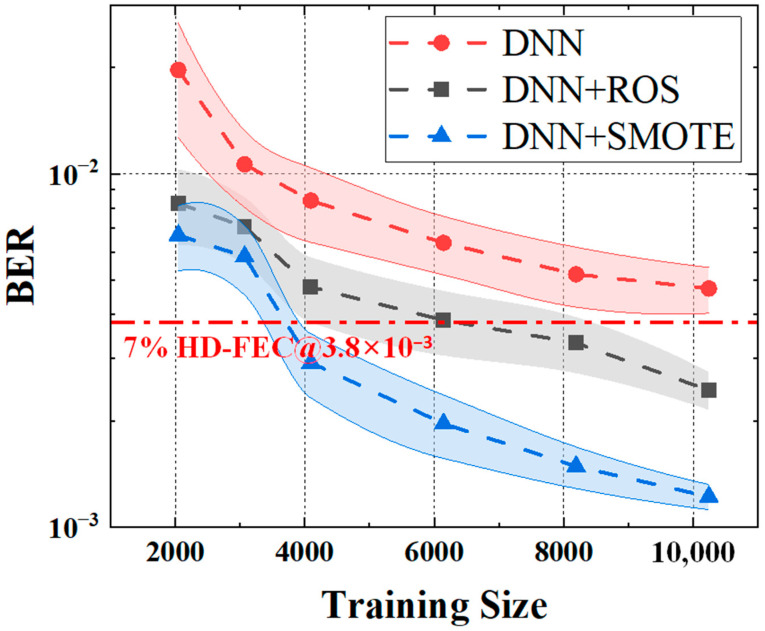
BER versus training sequence length for the DNN and the oversampling-based DNN.

In addition to evaluating the impact of the DNN architecture, we further investigate the impact of training sequence length on the BER performance of different equalizers, as shown in Figure 9. All models exhibit performance improvement as the training size increases from 2048 to 10,240, confirming that larger datasets benefit the equalization process. However, models incorporating oversampling techniques—especially SMOTE—achieve significantly better BER performance under the same training size compared to the baseline DNN. For instance, at a training size of 4096, the DNN + SMOTE equalizer achieves a BER of 3.57 × 10^−3^, successfully surpassing the HD-FEC threshold of 3.8 × 10^−3^, while the plain DNN equalizer remains above the threshold. Moreover, the DNN + ROS model only reaches this target when the training size exceeds 6144, whereas the DNN + SMOTE model already achieves this with 4096 samples—representing a 33% reduction in training data requirement. This confirms that SMOTE significantly improves the data efficiency of the learning process. These results demonstrate that incorporating synthetic data generation techniques such as SMOTE not only enhances the equalizer’s BER floor but also enables the model to reach performance saturation more quickly, thereby reducing the required dataset size and training complexity.

While the SMOTE-enhanced DNN demonstrates superior BER performance and robustness compared to ROS-DNN and conventional DNN, its potential drawbacks must also be considered. Specifically, the computational overhead of SMOTE is approximately an order of magnitude higher than that of ROS, which could become a bottleneck in real-time or resource-constrained environments.

Although SMOTE significantly reduces the number of floating-point operations (FLOPs) while maintaining similar bit error performance, its increased computational complexity may limit its applicability in scenarios that require real-time processing or have limited computational resources. Compared to the ROS method, SMOTE has a higher computational complexity. While SMOTE can help the equalizer achieve a lighter computational load, careful consideration is required to determine its suitability for practical applications. A balance must be struck between performance and computational complexity, particularly in real-time systems. The computational overhead of SMOTE could be mitigated through optimization techniques or by selectively applying SMOTE under specific conditions. Therefore, while SMOTE improves performance, its increased computational cost needs to be taken into account in resource-constrained or real-time processing environments.

### 4.3. Performance Comparison of Different Equalization Schemes

This section presents a comparative evaluation of four equalization schemes—CMA (Constant Modulus Algorithm), standard DNN, ROS-based DNN, and SMOTE-based DNN—in terms of BER performance and signal-level distributions when processing 3.75 Gbaud PAM signals under three shaping factors: V = 0.02, V = 0.05, and V = 0.1.

**Figure 10:**
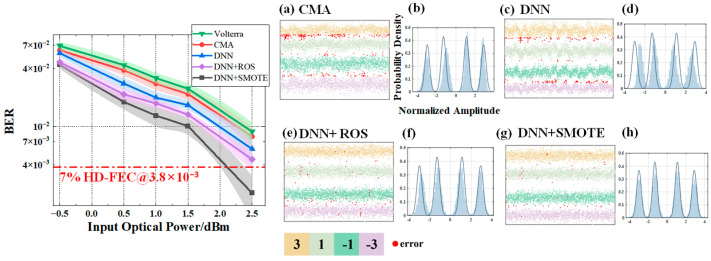
BER performance versus input optical power and signal levels under *V* = 0.02 for different equalizers: (**a**) CMA; (**c**) DNN equalizer; (**e**) ROS-based DNN equalizer; (**g**) SMOTE-based DNN equalizer. Subfigures (**b**), (**d**), (**f**), and (**h**) show the corresponding probability density distributions for (**a**), (**c**), (**e**), (**g**), respectively. Since the coordinate axes of (**b**,**d**,**f**,**h**) are identical, they are shown only in (**b**) for clarity.

**Figure 11:**
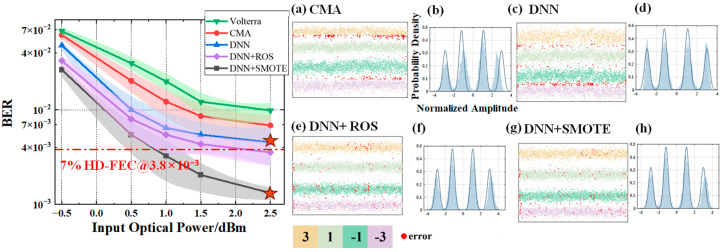
BER performance versus input optical power and signal levels under *V* = 0.05 for different equalizers: (**a**) CMA; (**c**) DNN equalizer; (**e**) ROS-based DNN equalizer; (**g**) SMOTE-based DNN equalizer. Subfigures (**b**), (**d**), (**f**), and (**h**) show the corresponding probability density distributions for (**a**), (**c**), (**e**), (**g**), respectively. Since the coordinate axes of (**b**,**d**,**f**,**h**) are identical, they are shown only in (**b**) for clarity. Stars denote the operating points with the largest BER improvement.

**Figure 12:**
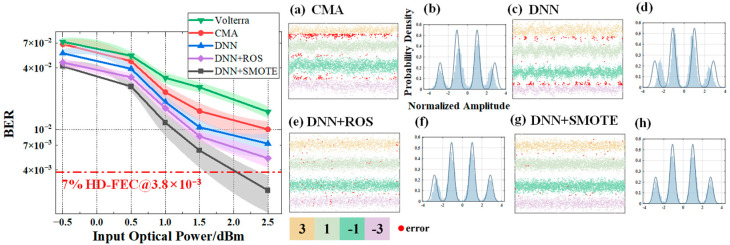
BER performance versus input optical power and signal levels under *V* = 0.1 for different equalizers: (**a**) CMA; (**c**) DNN equalizer; (**e**) ROS-based DNN equalizer; (**g**) SMOTE-based DNN equalizer. Subfigures (**b**), (**d**), (**f**), and (**h**) show the corresponding probability density distributions for (**a**), (**c**), (**e**), (**g**), respectively. Since the coordinate axes of (**b**,**d**,**f**,**h**) are identical, they are shown only in (**b**) for clarity.

In Figure 10, Figure 11 and Figure 12, subfigures (a–d) show the BER performance versus input optical power for the four equalizers, while subfigures (e–h) present their corresponding probability density distributions. All neural networks were designed with a single hidden layer to ensure a fair architectural comparison.

Figure 10 illustrates the results for V = 0.02, corresponding to a mild shaping condition with relatively weak channel nonlinearity. Across all equalizers, the BER decreases steadily as the input optical power increases from −0.5 dBm to 2.5 dBm, but noticeable performance differences remain. The SMOTE-based DNN equalizer achieves the lowest BER at all power levels, with average reductions of 45.04%, 32.24%, and 15.94% compared to CMA, standard DNN, and ROS-based DNN, respectively. The probability density plots confirm that SMOTE-based DNN produces more compact and well-separated clusters, indicating improved decision boundaries and reduced inter-symbol interference.

Building upon the results under *V* = 0.02, Figure 11 examines the case of *V* = 0.05, where moderate shaping introduces stronger nonlinear distortions. The performance gap between SMOTE-DNN and the other methods widens significantly. On average, SMOTE-DNN reduces BER by 64.87% compared to CMA, 48.11% compared to standard DNN, and 28.95% compared to ROS-based DNN. At 2.5 dBm, the BER drops from 4.52 × 10^−3^ (standard DNN) to 1.322 × 10^−3^ with SMOTE-based DNN, representing a 70.76% improvement. This demonstrates the robustness of SMOTE-based DNN in handling moderate nonlinearity while maintaining clear decision boundaries.

Finally, to assess performance under severe nonlinear distortion, Figure 12 presents the results for *V* = 0.1. Even in this challenging scenario, SMOTE-based DNN continues to outperform CMA and standard DNN by 45.97% and 32.73%, respectively. Although its BER is slightly higher than ROS-based DNN by 17.48% on average—likely due to localized fluctuations—it consistently surpasses the 7% HD-FEC threshold (BER = 3.8 × 10^−3^) at 2.5 dBm across all shaping factors. The probability density maps reveal that SMOTE-based DNN maintains better symbol clustering and sharper boundaries compared to CMA and standard DNN, evidencing its ability to mitigate the combined effects of nonlinearity and temporal jitter.

In summary, the SMOTE-based DNN equalizer significantly enhances nonlinear compensation capability under various shaping conditions and outperforms conventional and ROS-based approaches in both BER reduction rate and decision clarity. This makes it a compelling candidate for adaptive equalization in high-speed THz communication systems.

### 4.4. Inclusion of a Strong DSP Baseline for Performance Comparison

To provide a more credible evaluation of the proposed SMOTE-enhanced DNN equalizer, strong DSP baselines were introduced, including a second-order Volterra equalizer and a Constant Modulus Algorithm (CMA) equalizer. This enables a more rigorous assessment of performance improvements under comparable conditions, thereby demonstrating the effectiveness of the proposed method.

As a widely used nonlinear DSP technique, the Volterra equalizer employs second-order terms. Considering the algorithmic complexity and our goal of developing a lightweight equalizer, the number of taps was set to 1, making its performance comparable to that of an LMS equalizer. Under the same conditions, the CMA equalizer exhibits fewer residual errors and more reliable convergence, achieving superior equalization performance.

**Figure 13:**
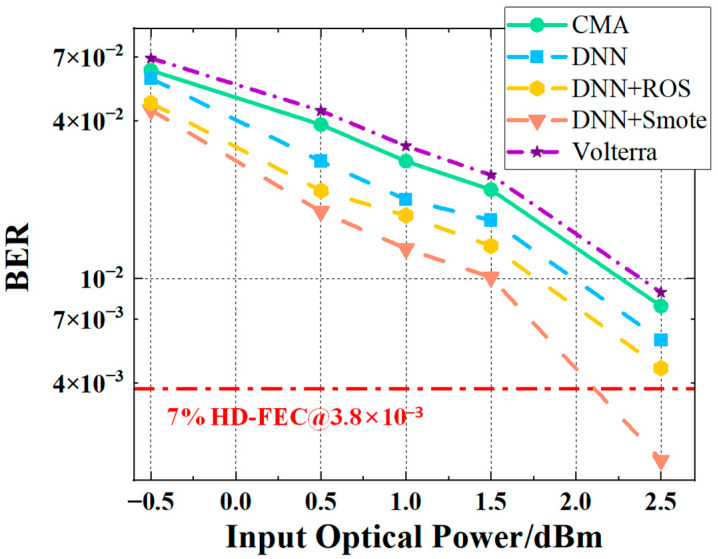
Bit Error Rate (BER) performance versus input optical power and signal levels under V = 0.02 for different equalizers, including CMA, DNN, DNN + ROS, DNN + SMOTE, and Volterra.

As shown in Table 3 and Figure 13, the computational complexities of the different equalization schemes are relatively similar. The FLOPs for DNN, DNN + ROS, and DNN + SMOTE are all 1170, while CMA has slightly lower FLOPs at 1157, and the Volterra equalizer has slightly higher FLOPs at 1230. Despite these differences, the overall computational complexities are comparable. However, the bit error rate (BER) performances differ significantly. DNN + SMOTE achieves the best BER, followed by DNN + ROS and DNN. In contrast, CMA and Volterra exhibit higher BER values, indicating that while the computational complexities are similar, substantial performance differences exist among the methods.

These results indicate that the introduction of SMOTE not only improves BER performance but also maintains a lightweight design, making the proposed equalizer superior in both performance and efficiency compared to conventional DNNs and traditional DSP baselines.

### 4.5. Performance Comparison of Different SMOTE Variants

**Figure 14:**
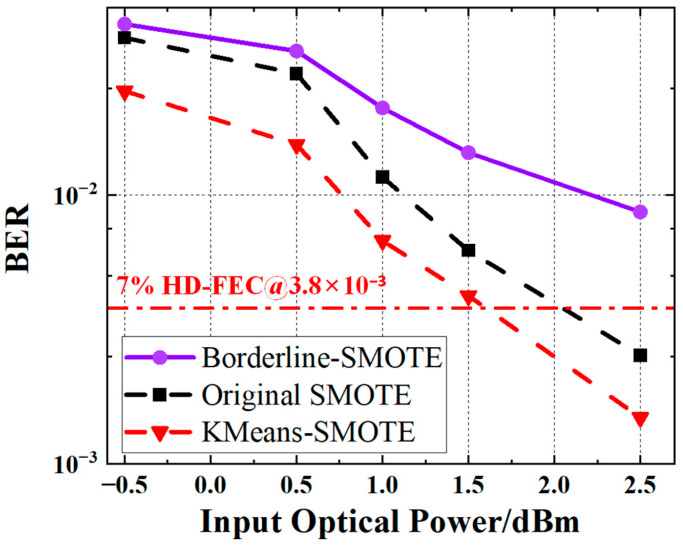
BER performance versus input optical power for PS-PAM4 signals using three SMOTE-based oversampling strategies.

To further investigate the impact of different oversampling approaches on equalization performance, we evaluate three representative SMOTE-based oversampling strategies—original SMOTE, Borderline-SMOTE, and K-Means-SMOTE—for PS-PAM4 signals. In addition, we compared the equalization performance of these methods under varying input optical powers ranging from −0.5 dBm to 2.5 dBm, as shown in Figure 14.

Overall, all three methods exhibited a clear downward trend in BER as optical power increased, although noticeable differences in performance were observed among the oversampling strategies. Among them, K-Means-SMOTE consistently outperformed the other two methods across all power levels. In the input power range of −0.5 dBm to 2.5 dBm, K-Means-SMOTE reduced the BER by 43.65% to 82.79% compared to Borderline-SMOTE, and by 36.62% to 41.11% compared to the original SMOTE. The performance gain of K-Means-SMOTE was particularly significant in the low optical power region (−0.5 dBm to 1.5 dBm), demonstrating superior BER performance. In contrast, Borderline-SMOTE performed the worst overall, with high BERs indicating that excessive focus on boundary samples may lead to imbalanced synthetic data distributions, thereby increasing model misclassification. The original SMOTE showed intermediate performance across all power levels, suggesting that while it effectively alleviates class imbalance, it does not optimize the data distribution structure, thus limiting its BER lower bound.

## 5. Conclusions

In this paper, we propose a lightweight DNN equalizer for THz RoF systems, integrating SMOTE oversampling and clustering-based adaptive thresholding to address class imbalance and MPI-induced amplitude jitter. Experiments on 3.75 Gbaud W-band PAM4 transmission over 300 m show that SMOTE-enhanced DNN achieves up to 70.7% BER reduction and 70.6% lower complexity than conventional equalizers, while reaching the HD-FEC threshold with 33% fewer training samples. Across shaping factors v = 0.02, 0.05, and 0.11, BER reductions reach 64.87% over CMA and 48.11% over baseline DNN, with K-Means-SMOTE delivering the best variant performance. These results highlight the method’s strong nonlinear compensation capability, computational efficiency, and robustness, making it well-suited for future 6 G optical-carrier-based wireless systems.

When considering the deployment of the algorithm on devices, especially on FPGA or DSP platforms, it is crucial to account for the computational complexity of the algorithm. Deploying neural networks on hardware platforms such as FPGAs often requires lightweight optimization techniques, such as pruning, quantization, and hardware-friendly architecture designs, to reduce memory and computational overhead while maintaining model performance.

In the proposed DNN equalizer, the integration of SMOTE significantly reduces computational complexity, making it more suitable for real-time applications. Specifically, SMOTE addresses the class imbalance issue, improving training efficiency and enabling the network to operate with fewer training samples. This results in faster inference speeds and lower resource usage. As demonstrated in our results, the reduction in computational complexity by 70.6% not only showcases the method’s efficiency but also lays the foundation for future hardware deployments, especially in resource-constrained real-time signal processing environments like FPGA/DSP.

In practical applications, the lightweight network structure achieved through SMOTE can be further optimized, for example, by pruning or quantization techniques, to tailor the DNN for specific hardware requirements, thereby further enhancing the feasibility of deploying the model on edge devices. This lightweight design makes our approach particularly promising for future 6G optical-carrier-based wireless systems, especially in real-time processing and low-latency scenarios.

## Figures and Tables

**Table 1:** Key Parameters of Experimental Components.

Component	Parameter
ECL 1	Wavelength: 1550.00 nm Linewidth: <100 kHz Output power: 14.5 dBm
ECL 2	Wavelength: 1551.03 nm Linewidth: <100 kHz Output power: 14.5 dBm
PD	Frequencyrange: 75–110 GHz
MZM	Bandwidth: 20 GHz
PA	Saturated output power: 13 dBm
LNA	Gain: 18 dB
HA	Gain: 25·dBi

**Table 2:** Summary of the power budget.

Parameter	Value
*P_T_*	2.5 dBm
*G_T_*	37 dBi
*G_R_*	55 dBi
*d*	200 m
*f*	82.5 GHz
FSPL	116.8 dB
*L*_AM_	0.14 dB
*P_R_*	−22.44 dBm

**Table 3:** Comparison of Equalization Scheme Complexity.

Network	Network Structure	FLOPs	BER
DNN	128-9-1	1170	5.83 × 10^−3^
DNN + ROS	128-9-1	1170	4.55 × 10^−3^
DNN + SMOTE	128-9-1	1170	2.03 × 10^−3^
CMA	231-1	1157	7.85 × 10^−3^
Volterra	(231,1)	1230	9.01 × 10^−3^

## Data Availability

The raw and processed data required to reproduce the findings of this study are not publicly available at this time, as they also form part of an ongoing extended study. Interested researchers may request access to the datasets from the corresponding author, Wen Zhou (email: zwen@fudan.edu.cn), subject to reasonable conditions and approval.

## Appendix A

**Algorithm A1:** SMOTE Algorithm

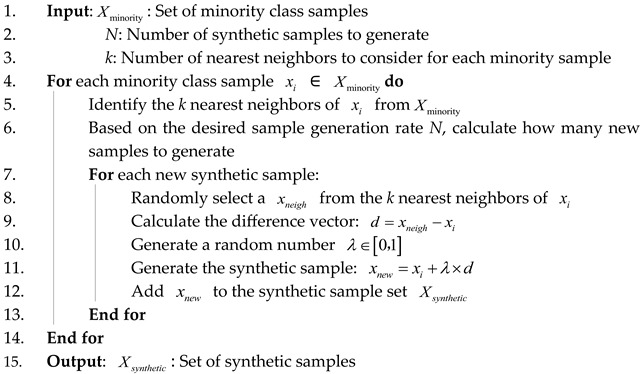

## Appendix B

**Algorithm A2:** DBSCAN Algorithm
